# Hypoalbuminemia as a severity predictor of preeclampsia in a multicenter study from Indonesia

**DOI:** 10.1038/s41598-026-55731-2

**Published:** 2026-06-08

**Authors:** Dina Marlina, Budi Handono, Aditya Utomo, Putri Nadhira Adinda Adriansyah, Siti Nadya Khairunissa Alamsyah, Herman Sumawan, Muhammad Alamsyah Aziz, Megawati Al’badly Ponco Dewi Poernomo

**Affiliations:** 1 Department of Obstetrics and Gynecology, Faculty of Medicine, Universitas Padjadjaran – Dr. Hasan Sadikin General Hospital, Jalan Pasteur no 38 Bandung, 40161 Bandung, Indonesia; 2 Department of Obstetrics and Gynecology, Faculty of Medicine, Universitas Jenderal Soedirman – Prof. Dr. Margono General Regional Hospital, Purwokerto, Indonesia; 3 Faculty Of Medicine, Universitas Jenderal Soedirman, Purwokerto, Indonesia

**Keywords:** Hypoalbuminemia, Preeclampsia, Predictor, Predictive markers, Biomarkers, Reproductive biology

## Abstract

Hypertensive disorders of pregnancy, affecting 5–10% of pregnancies, pose significant risks to maternal and fetal health. Pregnant women with preeclampsia and proteinuria experience a gradual decrease in serum albumin due to increased protein requirements, urinary protein loss, and endothelial damage. This study aimed to investigate the correlation between hypoalbuminemia and the severity of preeclampsia. A retrospective cohort study was conducted from 2022 to 2024 at Dr. Hasan Sadikin General Hospital and Regional General Hospital Prof. Dr. Margono Soekarjo. The study included 245 pregnant women diagnosed with preeclampsia who met specific inclusion criteria. Data were collected on age, body mass index (BMI), parity status, and albumin levels. Chi-square tests, relative risk, and receiver operating characteristic (ROC) curve analyses were utilized to assess the correlation between hypoalbuminemia and preeclampsia severity. Hypoalbuminemia was predominantly identified in individuals aged 31–40 years with mild to moderate hypoalbuminemia. Most participants were multiparous, obese, and presented with preeclampsia without severe features. A significant correlation (*p* = 0.000) was observed between hypoalbuminemia and preeclampsia severity. Pregnant women with severe hypoalbuminemia had a 4.4 times higher risk of developing preeclampsia with severe features compared to those with mild to moderate hypoalbuminemia. ROC analysis identified an albumin cut-off value of 2.51 g/dL as a predictor of preeclampsia with severe features. Hypoalbuminemia is significantly correlated with the severity of preeclampsia and may serve as a valuable predictor for identifying severe cases. Recognizing this relationship could improve the management of hypertensive disorders during pregnancy and potentially reduce maternal morbidity and mortality rates.

## Introduction

 Hypertensive disorders of pregnancy affect approximately 5–10% of pregnancies and can cause maternal, fetal, and neonatal morbidity and mortality^1^. Hypertension during pregnancy is defined as systolic blood pressure ≥ 140 mmHg and/or diastolic blood pressure ≥ 90 mmHg, measured on two separate occasions^2^. During normal pregnancy, blood pressure gradually decreases in the first trimester due to decreased systemic vascular resistance, reaches a low point at approximately 22–24 weeks, and then increases again from 28 weeks to preconception levels at 36 weeks of gestation^3^. Researchers have proposed a variety of hormonal, vascular, and metabolic factors to explain hypertensive disorders of pregnancy. Hypertension during pregnancy has a negative impact on women’s cardiovascular health later in life. According to a large population study, the prevalence of chronic hypertension during pregnancy has increased more than 13-fold in the last 4 decades (1970–2010)^4^.

Preeclampsia, with or without severe symptoms, is a pregnancy disorder characterized by new-onset hypertension, usually accompanied by proteinuria. It most often occurs after 20 weeks of gestation and is often close to the time of delivery. This disease covers the spectrum of hypertensive diseases in pregnancy, starting with gestational hypertension and progressing to severe symptoms, which can eventually lead to more severe manifestations, such as eclampsia and HELLP syndrome. Preeclampsia contributes to 2% to 8% of pregnancy-related complications, more than 50,000 maternal deaths, and more than 500,000 fetal deaths worldwide. Early diagnosis and quick treatment are needed to keep both the mother and the baby from having problems by managing symptoms and planning the delivery^5^.

There are two main subtypes of preeclampsia: early-onset preeclampsia (placental syndrome) and late-onset preeclampsia (maternal syndrome). Both have different etiologies and clinical manifestations^6^. In early-onset preeclampsia, the placenta has a problem that is linked to the start of preeclampsia. This is shown by the presence of placental infarction and vascular sclerosis. Abnormal trophoblast invasion leads to placental hypoperfusion, resulting in placental ischemia. On the other hand, maternal preeclampsia happens when a healthy placenta interacts with factors in the mother that damage small blood vessels. This damage may be caused by endothelial dysfunction in the mother^7^. Maternal preeclampsia, which occurs later in pregnancy, can be managed with expectant management until 37 weeks of gestation. In the late stages of pregnancy, maternal preeclampsia arises, requiring minimal changes in arterial conversion to maintain placental perfusion^5^.

Hypoalbuminemia and severe preeclampsia often occur together, and it has been suggested that hypoalbuminemia may be a marker of the severity of preeclampsia^8^. Hypoalbuminemia is a clinical condition characterized by a decrease in serum albumin levels below 3.5 g/dL^9^. Albumin is the major circulating protein that makes up 60% of the total plasma protein mass. Hypoalbuminemia causes a decrease in plasma oncotic pressure, which can result in extravasation, edema, and hypovolemia^10^. Low blood volume causes the kidneys to release more renin, which then raises the release of angiotensin I. This is then changed into angiotensin II (AII), which causes the release of aldosterone and higher blood pressure^11^.

During normal pregnancy, physiological hemodynamic changes occur, including decreased peripheral vascular resistance, reduced blood pressure, increased cardiac output, and plasma volume expansion. By term, approximately 6 to 8 L of fluid are retained across the fetus, amniotic fluid, and intracellular-extracellular spaces. As pregnancy progresses, serum albumin levels decrease due to plasma volume expansion and altered albumin metabolism^12,13^. In individuals with preeclampsia and proteinuria, serum albumin levels decline progressively due to increased protein demands, urinary protein loss, and translocation of albumin into the interstitial space caused by endothelial damage^12^. This progressive hypoalbuminemia may exacerbate fluid imbalance and contribute to worsening disease severity^12,13^.

Recognizing the potential link between hypoalbuminemia and preeclampsia severity, ongoing research seeks to explore this correlation to improve early detection and management of preeclampsia with severe features. This study aims to determine whether low albumin levels are associated with the severity of preeclampsia, thereby offering insights for better clinical management and prevention of adverse outcomes in pregnant women.

### Materials and methods

This was a cohort retrospective study conducted from 2022 to 2024 at Dr. Hasan Sadikin General Hospital (RSHS) and Regional General Hospital Prof. Dr. Margono Soekarjo (RSMS). Ethical clearance was obtained from Ethical Committee of Margono Hospital (Approval Number: 420/03289) and the Ethical Committee of Hasan Sadikin Hospital (Approval Number: DP.04.03/D.XIV.6.5/134/2024), and was carried out in accordance with the Declaration of Helsinki. Patients provided written informed consent for the use of their tissue samples in this research.

Pregnant women at any gestational age were enrolled in the study. Inclusion criteria include pregnant patients who gave birth at Dr. Hasan Sadikin General Hospital Bandung and Prof. Dr. Margono Soekarjo General Hospital Purwokerto in the period 2022–2024, diagnosed with preeclampsia, and medical records have complete supporting examination results for the diagnosis of preeclampsia. While exclusion criteria include patients with history of other infections (premature rupture of membranes, dental infections, respiratory infection such as tuberculosis and pneumoniae and other forms of infection), history of bowel surgery, history of chronic disease (renal disease, liver disease, diabetes, chronic hypertension), and Chronic Energy Deficiency (CED).

Preeclampsia without severe feature is a high blood pressure (≥ 140/90mmHg) and the presence of at least one of the following: proteinuria, acute kidney injury, neurological complications, hematological complications, or uteroplacental dysfunction Preeclampsia with severe feature is is a high blood pressure (≥ 160/110mmHg) and the presence of at least one of the following: thrombocytopenia (< 100.000), impaired liver function (more than twice upper limit normal level) or by severe right upper quadrant or epigastric pain unresponsive to medicine, renal insufficiency (Cr > 1.1 or doubling concentration in an absence other renal disease), pulmonary edema, new onset headache unresponsive to medicine, visual disturbance. Hypoalbuminemia is a clinical condition which is characterized by a decreased of albumin serum level below normal level (< 3.5 g/dL). In this study, albumin level was measured right after the patient’s diagnosed with hypertension, either chronic hypertension or hypertension which just developed in more that 20 weeks of pregnancy. Hypoalbuminemia was defined as a serum albumin concentration < 3.5 g/dL. For further stratification, patients were categorized into two groups: mild–moderate hypoalbuminemia (> 2.5–3.5 g/dL) and severe hypoalbuminemia (≤ 2.5 g/dL).

This study uses total sampling in the year 2022–2024 at Dr. Hasan Sadikin General Hospital (RSHS) and Regional General Hospital Prof. Dr. Margono Soekarjo (RSMS). The total number 4.290 pregnant women delivered that period, and after being excluded based on exclusion criteria, 245 total number of pregnant women were included as samples for this research. Chi-square was used to determine the correlation between the hypoalbuminemia and the incidence of preeclampsia. The receiver operating characteristic (ROC) curve was used to evaluate the ability of the albumin value to distinguish the preeclampsia with severe features. Chi-square and ROC curve was performed using the IBM SPSS Statistics version 25.0 (IBM Corp. Released 2017. IBM SPSS Statistics for Windows, Version 25.0. Armonk, NY: IBM Corp.).

Samples collected from the registered either pregnant women come from polyclinic or maternal emergency rooms who were hospitalized for giving birth. We used the following covariates: age, body mass index (BMI), parity status, and albumin value.

## Results

Table 1 summarizes the characteristics of the research subjects, including age, BMI, parity, severity of hypoalbuminemia, and severity of preeclampsia. The majority of participants were aged 31–40 years (49.8%), with the largest proportion falling into the obese category (46.9%) based on BMI. Parity status was relatively balanced across nullipara (33.5%), primipara (32.2%), and multipara (34.3%) groups.

Regarding hypoalbuminemia severity, most participants had mild to moderate hypoalbuminemia (69.0%), while 31.0% experienced severe hypoalbuminemia. In terms of preeclampsia severity, 60.8% of participants experienced preeclampsia without severe features, whereas 39.2% experienced preeclampsia with severe features. This distribution indicates that obesity and mild to moderate hypoalbuminemia are predominant characteristics within the study population.

**Table 1:** Characteristics of research subjects.

Patient characteristics	Preeclampsia without severe feature (*n* = 149)	Preeclampsia with severe feature (*n* = 96)	*p*-value
*Age*			0.001*
≤ 20	0 (0)	5(2)	
21–30	58 (23.7)	45 (18.4)	
31–40	77 (31.4)	45 (18.4)	
≥ 41	14 (5.7)	1 (0.4)	
*BMI*			0.735
Underweight (< 18.0 kg/m2)	11 (4.5)	7 (2.9)	
Normal weight (18.0–22.9 kg/m2)	42 (17.1)	21 (8.6)	
Overweight (23–24.9 kg/m2)	29 (11.8)	20 (8.2)	
Obese (≥ 25 kg/m2)	67 (27.3)	49 (19.6)	
Parity			0.159
Nullipara (0)	47 (19.2)	35 (14.3)	
Primipara (1)	44 (18)	35 (14.3)	
Multipara (≥ 2)	58 (23.7)	26 (10.6)	
Severity of hypoalbuminemia			0.000*
Mild - Moderate (> 2.5–3.5 g/dL)	137 (55.9)	32	
Severe (≤ 2.5 g/dL)	12 (4.9)	64 (26.1)	

Table 2 presents a cross-tabulation between hypoalbuminemia severity and the incidence of preeclampsia in pregnant women. Among those with severe hypoalbuminemia (*n* = 76), 64 individuals experienced preeclampsia with severe features, while 12 individuals experienced preeclampsia without severe features. In contrast, among those with mild to moderate hypoalbuminemia (*n* = 169), 32 individuals experienced preeclampsia with severe features, whereas 137 individuals experienced preeclampsia without severe features. This distribution suggests a notable association between severe hypoalbuminemia and the presence of severe preeclampsia.

**Table 2:** Cross tabulation between hypoalbuminemia and the incidence of preeclampsia.

Disease	Preeclampsia with severe feature (*n* = 96)	Preeclampsia without severe feature (*n* = 149)
Severe Hypoalbuminemia (*n* = 76)	64	12
Mild – moderate hypoalbuminemia (*n* = 169)	32	137

Table 3 demonstrates that pregnant women with severe hypoalbuminemia have a significantly increased risk of developing preeclampsia with severe features. The relative risk (RR) was calculated to be 4.45 [95% CI 3.039–8.673, p-value < 0.001], indicating that severe hypoalbuminemia increases the risk of severe preeclampsia by approximately 4.45 times compared to those with mild to moderate hypoalbuminemia.

**Table 3:** Correlation between hypoalbuminemia and the incidence of preeclampsia.

Severe hypoalbuminemia	Total	Outcome Variable	
		*N*	OR (95% CI)
Preeclampsia (*n* = 245)			
With severe features	96	64	4.45 (3.039–8.673)
Without severe features	149	12	

**Fig. 1:**
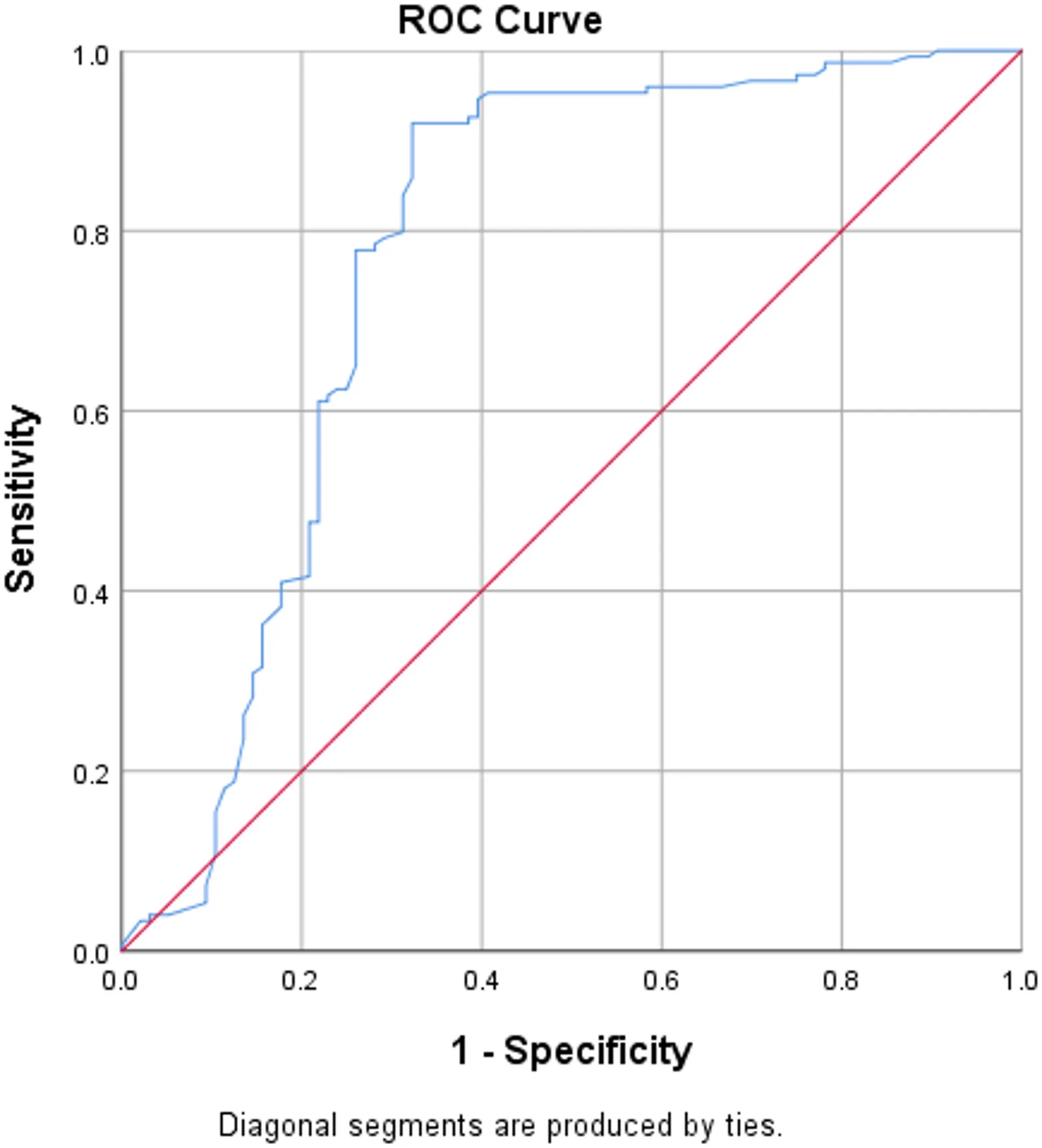
Hypoalbuminemia cut-off value to predict preeclampsia with severe features.

Figure 1 shows the ROC curve analysis for preeclampsia with severe features, with an area under the curve (AUC) of 0.772 [95% CI = 0.703–0.841], indicating good diagnostic accuracy. The optimal cutoff value determined by the Youden Index was a hypoalbuminemia level of 2.51 g/dL [OR. At this threshold, the sensitivity was 91.9% [95% CI = 0.869–0.956], and the specificity was 66.7% [95% CI = 0.569–0.756], indicating high sensitivity for detecting severe preeclampsia but with moderate specificity.

Table 4 demonstrates data analysis using multivariate logistic regression, conducted to assess the association between independent variables (degree of hypoalbuminemia, age, BMI, and parity) and the likelihood of the severity preeclampsia. Hypoalbuminemia was significantly associated with the severity of preeclampsia. In the crude model, women with hypoalbuminemia had higher odds of severe features of preeclampsia compared to the other group. After adjusting for potential confounders, the association remained significant. Among the model tested, the 2nd model demonstrated the best model fit with the lowest AIC (Akaike Information Criterion) value (27.884) and Nagelkerke R^2^ of 0.475, indicating that approximately 47.5% of the variability in preeclampsia severity was explained by hypoalbuminemia and the included covariates. AIC comparison favored model 2 over the crude and Model 1, suggesting better explanatory power with fewer predictors.

**Table 4:** Multivariable logistic regression models of the association of hypoalbuminemia and the severity of preeclampsia.

Levels	OR_crude_ (95%CI, *p*-value)	OR _model 1_ (95%CI, *p*-value)	OR _model 2_ (95%CI, *p*-value)
With severe features	22.833 (11.039–47.229, *p* < 0.001)	24.806 (11.098–55.444, *p* < 0.001)	21.823 (10.200–46.689, *p* < 0.001)
Without severe features	0.044 (0.021–0.091, *p* < 0.001)	0.040 (0.018–0.090, *p* < 0.001)	0.046 (0.021–0.098, *p* < 0.001)

Model description:

Crude: R^2^= 0.446; AIC = 230.321Model 1: Adjusted for age, body mass index, and parity. R^2^= 0.491; AIC = 105.233Model 2 (Model with better AIC): Adjusted for age, R^2^= 0.475; AIC = 27.884

## Discussion

The results of this study found that the majority of subjects with hypoalbuminemia were in the range of 30–41 years. This finding is supported by previous studies that found the average age of hypoalbuminemia with preeclampsia was 32.8 ± 4.1.5 Findings from other studies conducted in Indonesia also support this study, preeclampsia is dominated by pregnant women aged < 20 years and > 35 years, namely 58.3%^14^. This can be explained by the fact that as people age, their arteries tend to expand and become clogged, increasing the risk of severe conditions such as heart attacks or strokes. Additionally, the body’s ability to manage dietary salt declines with age, leading to poor dietary habits, physical inactivity, and reduced blood vessel flexibility, all of which ultimately contribute to hypertension in these individuals^15^.

In this study, it was found that the majority of pregnant women were obese. In the obese pregnant women, hypertension and chronic inflammation are the result of the release of cytokines from adipocytes^16^. The adipokines released include interleukin-1, interleukin-6, resistin and TNF-α (tumor necrotic factor-alpha), the latter two being directly involved in promoting insulin resistance. Other adipokines involved are leptin, whose release is increased, and adiponectin, the release of which is reduced. As adiponectin antagonizes Angiotensin-II, its decrease can explain, at least in part, the hypertension observed in the metabolic syndrome but also in pre-eclampsia in obese mothers. The increased circulation of inflammatory adipokines, in particular interleukin-1 and interleukin-6, give rise to multiple pathologies that share an inflammatory base. Among these pathologies, polycystic ovary syndrome, depression, infertility, and preeclampsia are the main clinical conditions associated with pregnancy^17^.

The results of the study related to the characteristics of parity status in pregnant women with preeclampsia, were found to be higher in the multiparous group than others. Pre-eclampsia frequently occurs in the initial pregnancy and typically does not reappear in later pregnancies. In primigravida, the development of blocking antibodies against placental antigens is less effective than in future pregnancies. Antibodies obstructing lead to maladaptation to the immune system, resulting in insufficient trophoblast invasion in the spiral arteries. The inability of trophoblast cells to invade the spiral artery walls prevents full dilation of the spiral arteries, leading to reduced blood flow in the intervillous region of the placenta and causing placental ischemia. This will release hazardous compounds that induce oxidative stress, a condition characterized by an excess of free radicals relative to antioxidants. This may lead to endothelial dysfunction affecting all endothelial surfaces of blood arteries in various organs. Endothelial dysfunction results in an imbalance in the synthesis of vasodilatory chemicals, leading to significant vasoconstriction and subsequent hypertension, which, if not diagnosed early, may progress to serious complications^18^. But it’s possible that the increased risk for the multiparous women in this study was confounded by other risk factors, including history of hypertensive disorders in the first pregnancy, co-existence of other complications, and unhealthy lifestyle. Future studies may be needed to examine the potential confounding factors, as well as the pathophysiology of pregnancy complications in women with different parity, to explore reasons behind this phenomenon^19^.

In this study, there was a correlation between hypoalbuminemia and the incidence of preeclampsia (*p* = 0.001). Hypoalbuminemia can be a predictor of severe preeclampsia at levels of 0.5 g/dL. Hypoalbuminemia is a comorbidity of preeclampsia and results from serum albumin leaking from the maternal body via urine^12^. Fetal-placental unit expends 1 kg of protein throughout the pregnancy approximately, mostly in the last 6 months. Diet containing 1.1 g/kg/day of protein is generally recommended to cover this need. This is usually higher than non-pregnant (0.8 g/kg/day)^20^. Serum albumin decreases across the normal pregnancy due to an increase in plasmatic volume and albumin metabolism. In the preeclampsia pregnant with proteinuria, a progressive decrease in serum albumin is present because of an increase of protein needs, loss of protein in urine and albumin pass to the interstitial liquid due to the endothelial damage. In these patients, an increase in sensitivity to angiotensin II is present, leading to vasoconstriction and increased blood pressure^13^.

The proposed cut-off value of 2.51 g/dL in our study should be interpreted in light of other thresholds that have been reported internationally. Several international studies have reported lower albumin cut-offs than our 2.51 g/dL threshold, underscoring the variability of albumin as a predictive marker in preeclampsia. Ciciu et al. found that a threshold of 2.3 g/dL achieved high sensitivity (~ 89%) and moderate specificity (~ 74%) for discriminating severe from mild preeclampsia. Albumin levels < 2.0 g/dL were significantly associated with severe proteinuria but weakly with other maternal or fetal complications, suggesting that very low albumin may reflect more the burden of protein loss rather than acting as an independent severity marker^21^. These variations indicate that the prognostic utility of albumin is not uniform across populations and may be influenced by differences in patient characteristics, severity of disease, and methodological approaches used to define outcomes.

Importantly, hypoalbuminemia in preeclampsia frequently reflects the combined effects of proteinuria, systemic inflammation, and endothelial dysfunction rather than an isolated pathogenic factor. Albumin is a negative acute-phase reactant, and its concentration can decline in the presence of systemic inflammatory activity, while significant urinary protein loss further contributes to reduced serum levels^22^. Therefore, although albumin can serve as a useful biomarker for identifying women at higher risk of severe disease, its predictive value is likely context dependent, and it should ideally be interpreted alongside other established clinical and biochemical indicators of disease severity such as the degree of proteinuria, renal function, and inflammatory markers^22,23^.

Albumin is an important indicator of the liver’s synthetic function and maintains the blood colloid osmotic pressure. Vascular endothelial cells are damaged in patients with preeclampsia and severe complications^24^, and the action of angiotensin increases vascular permeability leading to the loss of plasma proteins (especially Albumin) and a decrease in plasma colloid osmotic pressure that accelerates the appearance of intravascular lesions^12^. Previous studies found that the levels of Albumin in patients with gestational hypertension or chronic hypertension were lower than those in normal pregnant women^11,13,25^. In this study, we found that the levels of albumin in the preeclampsia group were significantly lower than those in the normal group.11 Now that we know the cutoff point for albumin levels in finding severe preeclampsia, we hope that serum albumin levels can be used as one of the signs of severe preeclampsia.

### Limitations

This study utilizes a retrospective cohort design, which may introduce bias due to its reliance on existing medical records. The accuracy and completeness of the collected data could be compromised, leading to potential misclassification of cases or incomplete information. Additionally, the research was conducted at only two hospitals, which may restrict the generalizability of the findings. Differences in clinical practices, diagnostic criteria, and patient demographics across various institutions could influence the results, limiting their applicability to broader populations.

## Conclusion

The conclusion drawn from this research is that monitoring serum albumin levels could serve as a valuable tool in identifying pregnant women at risk for severe preeclampsia, potentially guiding early interventions to mitigate maternal and fetal complications. Further studies are encouraged to explore this relationship in different populations and settings, aiming to improve management strategies for hypertensive disorders during pregnancy.

## Data Availability

The anonymized datasets generated and/or analyzed during the current study from both participating centers are available from the corresponding author upon reasonable request, in accordance with Scientific Reports’ open data policies.
